# Emerging strategies for durable Pt catalysts in PEMFCs

**DOI:** 10.1039/d5sc08221h

**Published:** 2026-01-27

**Authors:** Yuliang Chen, Linghang Meng, Haobo Sun, Honghong Lin, Shouheng Sun

**Affiliations:** a Department of Chemistry, Brown University Providence Rhode Island 02912 USA ssun@brown.edu; b Toyota Research Institute of North America 1555 Woodridge Ave. Ann Arbor Michigan 48105 USA

## Abstract

The performance and longevity of proton exchange membrane fuel cells (PEMFCs) are strongly influenced by the stability of platinum-based (Pt-based) catalysts. While significant progress has been made in enhancing catalytic activity, long-term degradation under harsh electrochemical conditions remains a critical challenge. This perspective reviews recent advances in understanding and mitigating the degradation mechanisms affecting Pt-based catalysts. It first examines key processes such as metal dissolution, catalyst poisoning, structural degradation, and carbon support corrosion that collectively impair catalytic performance. Next, it highlights emerging strategies to improve catalyst durability, including alloying, doping, and surface engineering techniques aimed at reinforcing both the Pt catalyst and its carbon support. Finally, it proposes potential pathways for the rational design of next-generation catalysts that combine high stability with superior efficiency for PEMFC applications.

## Abstract 2

Degradation mechanisms and stabilization strategies of Pt-based catalysts in proton exchange membrane fuel cells (PEMFCs).

## Graphical abstract

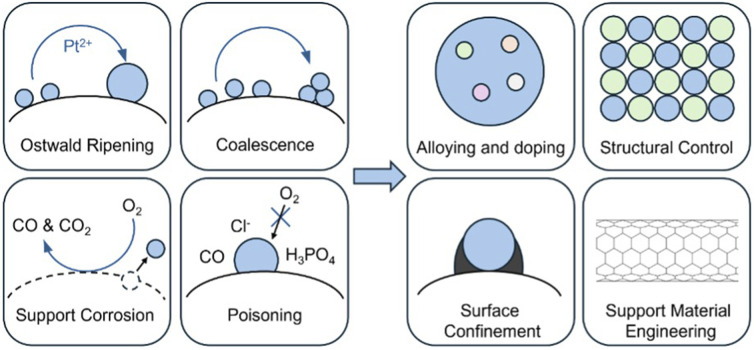

## 1 Introduction

The accelerating depletion of fossil fuel reserves, coupled with the escalating environmental consequences of carbon emissions, has driven the development of sustainable and clean energy technologies.^1,2^ Proton exchange membrane fuel cells (PEMFCs), also known as polymer electrolyte membrane fuel cells, have emerged as one of the most promising approaches to achieve efficient energy conversion.^3^ Due to their high energy efficiency, compact design, and potential for zero-carbon emissions, PEMFCs are regarded as an important green energy solution for decarbonizing the transportation, electronics, and power sectors.^4^

Over the past decades, substantial progress has been made to advance PEMFC technologies.^5^ Leading automotive manufacturers have commercialized fuel cell electric vehicles (FCEVs), such as Toyota Mirai and Hyundai NEXO, which offer driving ranges of 312–402 miles and reliable operation in temperatures as low as −30 °C.^6^ In California, approximately 50 fuel cell electric buses are currently in operation, with most meeting the durability benchmark of 25 000 hours under real-world driving conditions. At the same time, academic research continues to advance materials design, durability, and cost-effectiveness.^7^

A typical PEMFC consists of a membrane electrode assembly (MEA), comprising catalyst layers on gas diffusion electrodes (anode and cathode) separated by a proton-conducting polymer electrolyte membrane. The catalyst layers generally consist of platinum (Pt)-based nanoparticles (NPs) dispersed on a carbon black support, with an ionomer serving as both a proton conductor and a catalyst binder. The choice of membrane material depends largely on the operating temperature, which divides PEMFCs into two main categories: low-temperature PEMFCs (LT-PEMFCs) and high-temperature PEMFCs (HT-PEMFCs). LT-PEMFCs, typically operating at 60–95 °C, have a fast start-up, high specific power, and technological maturity.^8^ Perfluorosulfonic acid (PFSA) membranes, such as Nafion, are widely used for proton conduction. However, their proton conductivity strongly depends on the membrane's water content, posing significant challenges in terms of thermal and water management.^9^ Moreover, LT-PEMFCs exhibit lower tolerance to fuel impurities, as Pt catalysts suffer more severe poisoning at lower temperatures.^10^ By contrast, HT-PEMFCs operate at 120–250 °C, which enhances their tolerance to impurities, improves electrode kinetics, and simplifies thermal and water management.^11–13^ Different from LT-PEMFCs, HT-PEMFCs employ phosphoric acid (PA)-doped polybenzimidazole (PBI) membranes, where H_3_PO_4_ serves as the electrolyte to provide high proton conductivity.^14^ However, PA leaching and competitive phosphate adsorption on the Pt surface significantly hinder Pt catalytic performance.^15^

During PEMFC operation, hydrogen supplied to the anode undergoes oxidation *via* the hydrogen oxidation reaction (HOR), generating protons and electrons. The protons migrate through the electrolyte membrane to the cathode, while electrons travel through an external circuit to deliver electrical power. At the cathode, oxygen is reduced through the multielectron oxygen reduction reaction (ORR), combining with protons to form water. While the HOR is relatively fast and efficient, the ORR is kinetically sluggish, constituting the major bottleneck that limits overall cell performance and efficiency.^16^ Pt-based catalysts are widely adopted to catalyze PEMFC reactions, including HOR and ORR. Nevertheless, their high cost, limited natural abundance, and susceptibility to degradation under dynamic operating conditions remain significant barriers to the widespread commercialization of the PEMFC technologies.^17^ Since ORR requires higher Pt loadings than HOR, the cathode becomes the principal contributor to both system cost and performance degradation. Under realistic PEMFC conditions, especially during start–stop cycles and load fluctuations, insufficient electrochemical durability has emerged as a central challenge for Pt-based catalysts.^18^ Downsizing Pt catalysts into NPs effectively increases the electrochemically active surface area (ECSA) and improves mass-specific activity. However, the high surface energy of small NPs compromises their stability, promoting aggregation and coalescence, which diminishes ECSA and catalytic efficiency over time.^19,20^ Furthermore, Pt dissolution at high potentials can cause irreversible loss of active sites and redeposition on less active regions or the membrane, obstructing mass transport pathways and degrading overall performance.^21,22^ In addition, Pt NPs are vulnerable to poisoning by impurities such as sulfur and chloride ions, or leached phosphoric acid species in HT-PEMFCs, which block active sites and impair performance.^23,24^ These interconnected degradation pathways make it inherently challenging to balance high ORR activity with long-term durability. Consequently, extensive research has been directed toward improving Pt utilization and developing robust, active, and cost-effective catalyst architectures. Recent advances, including Pt-alloys, core@shell nanostructures, shape-controlled NPs, and single-atom catalysts, have demonstrated notable improvements in both catalytic activity and durability.^25–27^ While substantial progress has been made to meet the latest DOE M2FCT target for PEMFCs in HDV applications (1300 mA cm^−2^ at 0.7 V after 90k cycles),^28^ achieving further breakthroughs, however, requires a deeper understanding of the fundamental relationships between catalyst structure, surface properties, degradation mechanisms, and electrochemical performance in realizing PEMFCs as viable clean energy technology for the future.

In this perspective, we summarize recent progress in understanding the degradation and deactivation mechanisms of Pt-based cathode catalysts in PEMFCs and highlight emerging strategies aimed at enhancing their stability and durability. We first discuss the fundamental degradation mechanisms of Pt-based catalysts from multiple aspects, followed by a summary of recent approaches for designing durable Pt-based cathode catalysts. Finally, we offer our view on future research directions to further improve the durability and long-term performance of Pt-based catalysts in practical fuel cell systems.

## 2 Degradation and deactivation mechanism of Pt-based catalysts

### 2.1 Metal dissolution and degradation

To enhance the intrinsic ORR activity, Pt alloys with first-row transition metals such as Co, Fe (*e.g.*, PtCo, PtFe) have been widely employed as advanced Pt-based catalysts.^29,30^ In PEMFCs, however, the cathode environment is strongly acidic. Under such conditions, transition metals in Pt alloys are unstable and tend to dissolve into the electrolyte. The dissolved metal ions can migrate through the membrane and redeposit on the anode catalyst, covering the anode catalyst and thus lead to degradation of overall cell performance. Moreover, the leaching of alloying elements disrupts the ordered alloy lattice and induces surface reconstruction, which further accelerates Pt dissolution and performance loss.^31–33^

The dissolution of Pt itself during ORR represents a major degradation pathway for Pt-based catalysts. The oxidation of metallic Pt^0^ to soluble Pt^2+^ or Pt^4+^ species leads to loss of ECSA and decreased ORR activity. Additionally, dissolved Pt species can migrate and redeposit at the cathode/membrane interface, blocking mass-transport channels and increasing oxygen transport resistance.^34^*In situ* studies using gas diffusion electrode (GDE) setups coupled with inductively coupled plasma mass spectrometry (ICP-MS) have enabled time-resolved monitoring of Pt dissolution during potential cycling.^35^ These studies reveal that Pt dissolution occurs in both anodic and cathodic scans, with higher dissolution rates being observed during the initial anodic sweep due to oxidative dissolution of undercoordinated surface sites.^20^ The oxide formed during anodic polarization has been identified as an interconnected square-planar PtO_4_ network, structurally resembling bulk Pt_3_O_4_. Upon reduction, Pt_3_O_4_ transforms into the soluble species PtOH(H_2_O)_3_^2+^, which can further hydrate to yield Pt(H_2_O)_4_^2+^.^21^ Recent *in situ* high-energy surface X-ray diffraction studies have demonstrated that anodic and cathodic dissolution processes are associated with distinct surface oxides. Anodic dissolution correlates with the formation of stripe-like PtO_*x*_ species, whereas cathodic dissolution is linked to amorphous PtO_2_ formed after surface oxide saturation.^36^

**Fig. 1:**
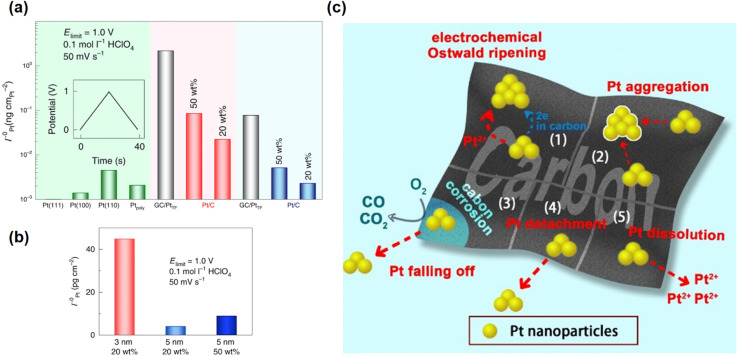
(a) Pt dissolution from Pt (111), (100), (110) and polycrystalline Pt, Pt(poly), surfaces (green bars), Pt NPs of 3 nm (grey bars, red background) or 5 nm (grey bars, blue background) supported on glassy carbon (GC), Pt/C NPs of 3 nm with 20 or 50 wt% loading (red bars) and those of 5 nm with 20 or 50 wt% loading (blue bars). (b) Amount of Pt dissolved from different Pt catalysts. The amount was obtained by integrating the dissolution curves of a single anodic and cathodic scan cycle. Adapted from ref. 20 with permission. Copyright © 2020, Springer Nature. (c) The performance loss mechanism of Pt/C catalyst in PEMFC. Reproduced from ref. 42 with permission. Copyright © 2023, The Royal Society of Chemistry.

Pt dissolution kinetics are dependent on many factors. When the catalyst surface is populated with undercoordinated or strained Pt sites, fast Pt dissolution occurs. For instance, the low-coordination Pt(110) surface dissolves faster than the more stable Pt(111) facet (Fig. 1a).^37^ Similarly, smaller NPs (∼3 nm) exhibit higher fractions of corner and edge atoms, resulting in up to ninefold faster dissolution compared to larger (∼5 nm) Pt NPs (Fig. 1b).^20^ The applied potential window can also affect the Pt dissolution behavior: in the accelerated durability testing, a wider potential range (0.4–1.0 V) leads to faster Pt dissolution compared to the narrower window (0.6–0.95 V), indicating that both oxidation and reduction processes contribute to Pt loss.^38^ The apparent dissolution rate depends on the mobility of Pt ions and the local electrolyte environment.^39^ Catalysts with higher Pt loading (Fig. 1b), or denser three-dimensional (3D) porous structures, or even different MEA configurations, exhibit lower apparent dissolution rates due to limited ion transport.^20,35^ The coupled dissolution and redeposition of Pt ultimately lead to catalyst coarsening. One dominant pathway is Ostwald ripening, where Pt atoms dissolve from smaller or more active particles and redeposit onto larger ones, resulting in particle growth.^40^ Pt NPs may also migrate and coalesce with neighboring particles^41^ or detach from the corroded carbon support, resulting in catalyst loss (Fig. 1c).^42^ These Pt dissolution and redeposition steps reduce the overall ECSA and accelerate its degradation in PEMFCs.

### 2.2 Pt poisoning

The poisoning of Pt refers to the occupation of Pt active sites by strongly adsorbed species, which can originate from either the feed gas or the cell assembly, including sealing gaskets, bipolar plates, membranes, or cooling systems.^43^ Such poisoning inhibits Pt catalysis for the ORR and significantly deteriorates the performance of PEMFCs.

The ORR occurring at cathode electrodes relies on a continuous supply of O_2_ from air. The reaction efficiency is strongly dependent on the air purity. Atmospheric air may contain contaminants such as NO_*x*_, CO, and sulfur-containing species (SO_2_, H_2_S) that can all bind to Pt strongly. This strong adsorption blocks active sites from accessing O_2_ and, as a result, reduces ORR activity.^44–46^ The poisoning may further shift the ORR mechanism. For example, high concentration of SO_2_ coverage can shift the reaction pathway from the 4e^−^ ORR to the 2e^−^ reduction process, yielding H_2_O_2_, which is detrimental to the Nafion binder in the catalyst layer.^47^

Pt cathodes can also be poisoned by halides (Br^−^, Cl^−^, I^−^), which are often present in catalyst precursors or in ambient air, particularly in coastal regions. Halides can bind to Pt more strongly than O_2_, reducing the number of effective active sites for ORR.^48,49^ The poisoning kinetics follow the trend F^−^ < Cl^−^ < Br^−^ < I^−^. At halide concentrations as low as 0.02 ppm, Pt/C exhibits ECSA losses of 47%, 49%, 56%, and 97% for F^−^, Br^−^, Cl^−^, and I^−^, respectively.^23^ This loss results not only from site blocking but also from halide-promoted Pt dissolution, which accelerates particle aggregation *via* Ostwald ripening.^23,50^

Another possible source of catalyst poisoning in LT-PEMFCs is the ionomer used as a binder in the catalyst layer. PFSAs, such as Nafion, are widely employed as ionomers.^51^ The sulfonate/sulfonic acid groups (–SO_3_^−^/–SO_3_H) in PFSA can bind to Pt under oxidative potentials (0.4–0.6 V), blocking oxygen access and suppressing ORR kinetics.^52^ Sulfonate adsorption is proposed as a one-electron transfer process (–SO_3_^−^ + Pt → –SO_3_–Pt + e^−^) with Pt–O bond formation.^53^ Adsorption strength is influenced by both the cationic environment and the polymeric backbone. Desorption requires overcoming electrostatic interactions between sulfonates and cations, proceeding *via* a coupled cation–electron transfer. Adsorbed sulfonates act as site blockers and modify the adsorption behavior of OH/O species on adjacent Pt sites. Additional adsorbates associated with the Nafion binder (*O_Nafion_ and *OH_Nafion_) have been identified, and a sluggish transition from *O to *OH is proposed as a key factor suppressing ORR activity.^54^ Oxygen atoms in ether groups can also interact with Pt, but restricted side-chain flexibility can alleviate ORR suppression.^53^*In situ* studies indicate that sulfonic acid groups can degrade on fluorocarbon backbones, forming sulfate species (SO_4_^2−^), which can be reduced to sulfite (SO_3_^2−^) or sulfide (S^2−^) species, further contributing to Pt poisoning.^55^

Phosphoric acid presented in the HT-PEMFCs can strongly bind to Pt *via* Pt–O–P bonds, deactivating the catalyst. This adsorption decreases ECSA and inhibits ORR kinetics,^15,56^ necessitating higher Pt loadings compared to H_3_PO_4_-free LT-PEMFCs.^57^ H_3_PO_4_ adsorption occurs within ∼0.3–0.8 V, between hydrogen and oxygen adsorption potentials,^24^ and is sensitive to pH, concentration, and temperature. High concentrations favor atop or bridge adsorption, while dilute concentrations favor threefold inverted geometries.^58^ Δ*µ* X-ray Absorption Near Edge Structure (XANES) analysis indicates reduced H_3_PO_4_ surface coverage at elevated temperatures, where atop/bridge geometries dominate at low temperatures and threefold inverted configurations dominate at high temperatures.^24^ H_3_PO_4_ competes with *OH for Pt–O bonding, shifting the onset potential for *O and *OH formation and increasing ORR overpotential. Theoretical studies suggest H_3_PO_4_ mainly acts as a site blocker without altering the intrinsic activity of adjacent Pt sites.^59^ In addition, H_3_PO_3_, formed by H_3_PO_4_ reduction at the anode or present as impurities,^60^ shows stronger Pt affinity, further suppressing ORR. DFT calculations indicate preferential pyramidal adsorption on Pt(111) *via* Pt–P bonds, which is supported by Δ*µ* XANES *in situ* X-ray adsorption spectroscopy (XAS) experiments.^61^ At potentials >0.7 V or elevated temperatures, H_3_PO_3_ can be oxidized to H_3_PO_4_ by H_2_O or PtO_*x*_, partially alleviating poisoning.^61,62^

### 2.3 Support corrosion

Pt-based nanocatalysts are commonly supported on carbon black materials owing to their excellent electrical conductivity and high specific surface area. Carbon blacks such as Vulcan XC72, Acetylene Black, and KetjenBlack (KB) are widely employed as catalyst supports in PEMFCs. However, the carbon species in these supports are prone to oxidation or corrosion, particularly under high humidity, strong acidity, and elevated temperature conditions.^63^ The mechanism of carbon corrosion generally proceeds through a two-step process driven by a cathodic potential. In the first step, carbon is oxidized by H_2_O to form surface oxygen-containing functional groups, as revealed by *in situ* attenuated total reflectance Fourier transform infrared (ATR-FTIR) spectroscopy.^64^ These surface oxides are subsequently oxidized to gaseous CO and CO_2_.^65^ The overall reactions can be represented in eqn (1) and (2).

1 C + H_2_O → CO + 2H^+^ + 2e^−^ (0.518 V *vs.* RHE)

2 C + 2H_2_O → CO_2_ + 4H^+^ + 4e^−^ (0.207 V *vs.* RHE)

A high cathode potential serves as a major driving force for carbon oxidation, especially under open-circuit conditions or during frequent start–stop cycling when the potential exceeds 1.0 V *versus* SHE.^66^ It has been reported that carbon corrosion can occur at room temperature when the cathode potential is above 1.0 V,^67^ and that every 0.1 V increase above this threshold accelerates the corrosion rate by roughly an order of magnitude.^68^ The corrosion rate strongly depends on the structural characteristics of the carbon support. Carbons with larger Brunauer–Emmett–Teller (BET) surface areas tend to suffer higher weight loss during corrosion,^69^ while amorphous carbons are generally more susceptible than well-graphitized counterparts.^70^ Pt can also catalyze carbon corrosion by facilitating the formation of reactive oxygen intermediates that promote carbon oxidation at lower potentials (0.1 V *vs.* RHE).^71^ More detailed studies indicate that Pt mainly catalyzes the initial oxidation of disordered carbon regions while potentially suppressing further corrosion of more ordered sites.^72^

Carbon corrosion compromises the integrity of the support structure, leading to Pt NP agglomeration or detachment, a significant reduction in ECSA, and severe deterioration in cell performance. Consequently, alongside efforts to stabilize Pt, considerable attention is being directed toward developing more robust and corrosion-resistant catalyst supports for next-generation fuel cells.

## 3 Strategies to enhance stability/durability of Pt-based catalysts

### 3.1 Alloying and doping

Alloying has long been an effective strategy to enhance both the activity and poisoning resistance of Pt-based catalysts. Alloying Pt with common transition metals such as Fe, Co, Ni, and Cu can lead to a downshift of the Pt d-band center, primarily due to the strain effect induced by smaller atomic radii and the ligand effect arising from electronic interactions. This downshift weakens the adsorption strength of oxygenated intermediates (*e.g.*, *O and *OH), thereby improving the adsorption–desorption equilibrium and enhancing the ORR activity.^73^ Alloying Pt with lanthanide-group metals introduces the well-known lanthanide contraction effect, which imposes localized compressive strain on the Pt lattice, also resulting in a downshift of the d-band center and enhanced ORR activity.^74^ This alloying-induced downshift in the d-band center leads to weaker adsorption of poisoning species, such as sulfur-containing species, phosphoric acid, and CO.^75–77^*Operando* X-ray photoelectron spectroscopy (XPS) revealed that PtCo alloys exhibit weaker adsorption toward S^2−^, SO_3_^2−^, and SO_4_^2−^ compared with pure Pt.^75^ Similarly, Pt_3_M/C catalysts (M = Ni, Co, Fe) show reduced adsorption of phosphoric acid due to the alloying-induced downshift of the d-band center.^76^ Cu is added into the L1_0_-PtFe structure to modulate lattice strain, weakening phosphoric acid adsorption while simultaneously improving ORR activity.^78^

Although alloying with early transition metals and rare earth elements offers advantages in enhancing both ORR activity and resistance to poisoning, it often compromises catalyst stability in acidic environments due to the leaching of the transition metals themselves, leading to loss of their beneficial effects. Interestingly, increasing the number of alloying elements can significantly enhance resistance to metal dissolution, as demonstrated by recently developed high-entropy alloys (HEAs). HEAs, typically composed of more than five elements, have emerged as a promising class of Pt-based cathode materials.^73,79^ Their exceptional properties arise from several unique effects: high entropy, lattice distortion, sluggish diffusion, and the cocktail effect, which synergistically enhance ORR performance.^79^ Improved stability mainly originates from the high-entropy and sluggish-diffusion effects, while the others primarily influence electronic structure and catalytic activity. Specifically, the high-entropy effect arises from the large configurational entropy of multielement mixing, stabilizing single-phase solid solutions and preventing phase segregation.^80^ This thermodynamic stabilization improves NP robustness during high-temperature processing. Meanwhile, the disparity in atomic radii among constituent elements induces asymmetric bonding and significant lattice distortion, which reduces atomic mobility (the sluggish diffusion effect),^81^ thereby suppressing metal dissolution and enhancing structural stability under fuel-cell operating conditions. For example, the energy barrier for Pt vacancy formation, an essential step in Pt diffusion and dissolution, is much higher in PtFeCoNiCu HEA NPs than in binary PtNi alloys, likely due to the localized lattice strain introduced by additional alloying elements.^82^ These findings highlight alloying as an effective approach to suppress the dissolution of both early transition metals and Pt in PEMFC cathodes.

**Fig. 2:**
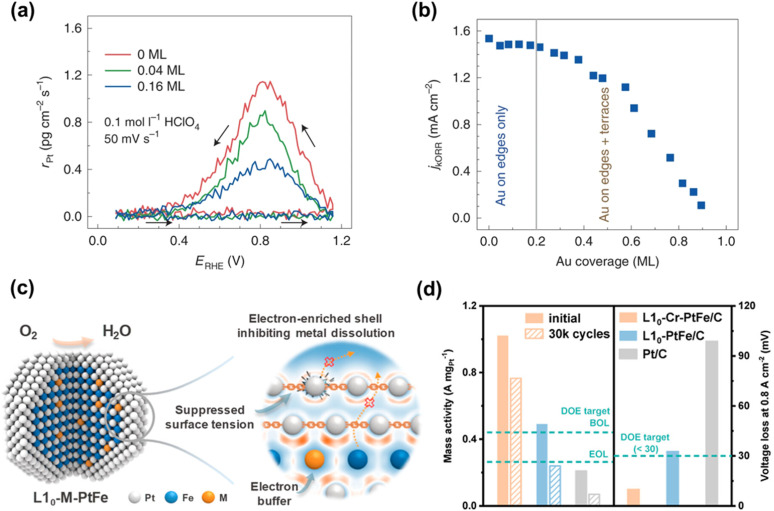
(a) Pt dissolution rate for Pt(111) covered by Au at 0.04 and 0.16 ML. The Pt contents were measured by a stationary probe rotating disk electrode ICP-MS (SPRDE-ICP-MS) in the first cycle up to 1.15 V. (b) ORR kinetics of Pt surface covered by different layers of Au (*j*_k_ measured at 0.9 V in 0.1 mol l^−1^ HClO_4_ at 50 mV s^−1^ and 1600 r.p.m.). Reproduced from ref. 20 with permission. Copyright © 2020, Springer Nature. (c) Illustration of the improvement mechanism of electron buffers in inhibiting metal dissolution of the L1_0_-M-PtFe catalyst. (d) Mass activity retention and voltage loss at 0.8 A cm^−2^ of the L1_0_-Cr-PtFe before and after ADT. Reproduced from ref. 83 with permission. Copyright © 2024 American Chemical Society.

To mitigate metal dissolution further, additional doping strategies have been explored. Incorporating acid-stable metals such as Au or Rh helps to prevent both Pt and transition-metal dissolution. For instance, small amounts of Au deposited on Pt surfaces preferentially occupy step and edge sites. When the Au surface coverage increases from 0.04 to 0.16 monolayers (ML), the Pt dissolution rate decreases threefold due to the blocking of these high-energy sites (Fig. 2a). Further increasing Au coverage has minimal additional effect on dissolution but begins to hinder ORR activity because of excessive site blocking. Notably, sub-0.2 ML Au coverage preserves ORR activity while significantly enhancing stability (Fig. 2b).^20^ Rh doping has also been shown to strengthen Pt–Pt bonding, suppressing Pt dissolution and protecting transition metals in the alloy core.^84–86^

Doping earlier transition metals such as Ti, V, Cr, and Nb into the L1_0_-PtFe structure can create an electronic buffer to protect Pt against oxidation (Fig. 2c).^83^ For example, Cr doping in the L1_0_ structure has a pronounced electron-donating effect, enriching the Pt shell and thereby enhancing its resistance to oxidative degradation. Strong Cr–Fe interactions also increase the energy barrier for Fe diffusion—a key step in Fe dissolution. Consequently, Cr-doped L1_0_-PtFe NPs exhibit only a 10 mV voltage loss at 0.8 A cm^−2^ and negligible compositional change after 30 000 accelerated durability test (ADT) cycles in MEAs, whereas undoped L1_0_-PtFe suffers a 33 mV loss with significant Fe dissolution (Fig. 2d).^83^ This “electron-buffer” concept was extended to a broader range of 4th-period elements (Ti to Ge). When these elements are alloyed with L1_0_-PtM (M = Fe, Co, Ni), metallic bonds partially transform into quasi-covalent networks due to reduced antibonding-state occupancy associated with high-lying d-band centers of the dopants. This strengthens atomic bonding and suppresses metal dissolution. For instance, L1_0_-PtCoCr exhibits only a 5 mV voltage loss at 0.8 A cm^−2^ with negligible compositional change.^87^

**Fig. 3:**
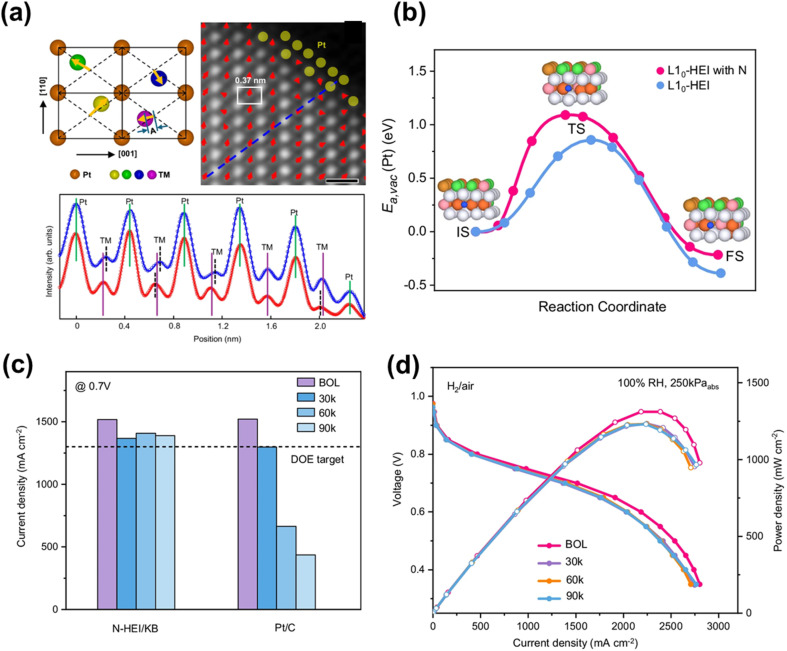
(a) Schematic of the sub-angstrom displacement of transition metals and the STEM-HAADF image from part of an N-doped HEI NP. The line scan profile below shows the displacement of transitional metals in N-doped HEI (blue: N-doped HEI; red: HEI). (b) The diffusion barrier path and activation energy of Pt vacancy for L1_0_-HEI with/without N-doping using DFT calculations. (c) Current density of N-doped HEI/Ketjenblack and commercial Pt/C at 0.7 V under different ADT cycles. (d) H_2_/air fuel cell performance of N-doped HEI/Ketjenblack at the beginning of life (BOL) and different voltage cycles, 0.20mg_Pt_ cm^−2^ (cathode Pt loadings), H_2_/air (500/2000 sccm), 80 °C, and 250 kPa_abs_ pressure. Reproduced from ref. 91 with permission. Licensed by CC BY 4.0.

Doping Pt with p-block elements (*e.g.*, B, N, P) has also proven effective for improving its electrochemical durability by enhancing d–p orbital interactions within Pt. For example, B-doped Pt shows only a 15% activity decay after 30 000 ADT cycles, compared with a 45% decay for pure Pt.^88^ The improved durability is attributed to B–Pt interactions that weaken Pt–O/Pt–OH bonding, thereby increasing oxidation resistance. N doping in L1_0_-PtNi has been shown to form strong Ni–N bonds that induce a “pinning effect,” preventing leaching of Ni and stabilizing the structure that is more stable than L1_0_-PtNi.^89^ This concept has been extended to HEA systems: for example, N-doped PtNiFeCoCu alloys stabilize core transition metals through multiple M–N bonds.^90^ Beyond the pinning effect, N doping introduces lattice distortion (Fig. 3a), increasing diffusion barriers for both Pt and transition metals (Fig. 3b), further enhancing resistance to dissolution and aggregation. Such catalysts exhibit only a 9 mV loss at 0.8 A cm^−2^ and maintain a current density of 1388 mA cm^−2^ at 0.7 V after 90 000 ADT cycles in MEAs under HDV conditions (Fig. 3c and d).^91^ Compared with B or N doping, P doping does not exhibit a comparable enhancement effect.^92^

Finally, doping with rare-earth elements such as Y, La, Ce, and Gd has also shown promise for improving Pt-based catalyst durability. These oxophilic elements readily form stable M–O species during ORR, which weakens Pt–O bonding and thereby reduces oxidative dissolution. For instance, Gd–O dipoles introduced into Pt_3_Ni helped retain 72.1% of its mass activity with minimal aggregation after 70 000 ADT cycles in an RDE setup, compared to only 40.7% retention for Pt_3_Ni.^93^ Similarly, Y-doped PtCo catalysts exhibited improved durability due to Y–O formation and strong Y–Co interactions, which increased vacancy formation energies for both Pt and Co, suppressing overall metal dissolution.^94^ However, excessive incorporation of rare-earth elements may compromise catalyst stability, as these metals are themselves prone to dissolution under acidic conditions.^95^

### 3.2 Structural engineering: intermetallic and core@shell structure

Although alloying significantly improves intrinsic ORR activity, early transition metals on the NP surface inevitably undergo dissolution during operation, a Pt-rich shell generally forms *in situ*, protecting the alloy core from further leaching. This observation has inspired the deliberate construction of core@shell structures as a widely adopted strategy to simultaneously enhance catalyst activity and durability. While PtM (M = Co, Fe, Ni) alloys are prone to M-metal leaching, thereby losing the beneficial electronic effects of alloying, the formation of an acid-resistant Pt shell can prevent sublayer metal dissolution while preserving the favorable electronic modulation imparted by the underlying alloy. This results in improved ORR kinetics and enhanced tolerance to poisoning species. Extensive studies have focused on fabricating Pt alloy core@Pt-shell structures.^96^

Intermetallic Pt-based cathode materials, characterized by their long-range atomic ordering and well-defined stoichiometry, have emerged as a promising class of catalysts with enhanced stability and activity for fuel cell applications.^73^ The most common intermetallic structures used for ORR catalysis are binary Pt-based compounds, typically denoted as PtM (M = Co, Fe, Ni, Cu), which adopt either the L1_0_ (PtM) or L1_2_ (Pt_3_M) ordered phases. Owing to the strong 3d–5d electronic interactions between M and Pt, the formation enthalpy of Pt–M intermetallics is highly negative, resulting in stronger Pt–M bonding. This stabilizes the alloy structure, rendering it more resistant to dealloying in acidic environments and more durable under PEMFC operating conditions.^97^ For example, L1_0_-PtCo exhibits only ∼10% Co dissolution, whereas disordered A1-PtCo shows ∼68% Co leaching after immersion in 0.1 M HClO_4_ at 60 °C for 24 h.^29^ The long-range atomic ordering in intermetallics also amplifies strain effects, which can further enhance ORR activity and improve tolerance to poisoning species. This is evidenced by the stronger phosphoric acid resistance observed for intermetallic PtCu compared with disordered PtCu.^98^

**Fig. 4:**
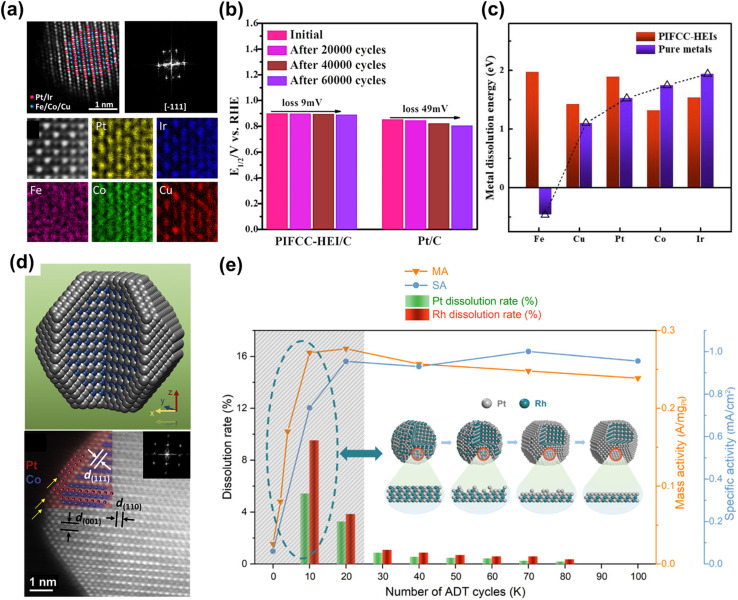
(a) HAADF-STEM image and corresponding EDS elemental mapping of PtIrFeCoCu HEI NPs, showing the ordered intermetallic structure. (b) Comparisons of the half-wave potentials of HEI/C and commercial Pt/C before and after different cycles of ADTs. (c) Comparison of the metal dissolution energy of HEI NPs and the pure metals. PIFCC: PtIrFeCoCu. Reproduced from ref. 100 with permission. Copyright © 2023, American Chemical Society (d) schematic illustration and STEM image of L1_0_-CoPt@Pt NPs with 2–3 atomic layers of Pt shell over L1_0_-CoPt core. Reproduced from ref. 29 with permission. Copyright © 2018 Elsevier Inc. (e) Mass activity and specific activity of PtRh NPs/C and dissolution rate of Pt and Rh. The inset schematic shows the ‘self-healing’ mechanism during ADT cycles. Adapted from ref. 102 with permission. Copyright © 2022, American Chemical Society.

Building upon binary intermetallics, high-entropy intermetallics (HEIs) have recently emerged as a new class of Pt-based catalysts that further enhance the chemical and structural robustness of cathode materials. Conversion of HEAs into HEIs increases both the mixing enthalpy and atomic interaction strength relative to their disordered counterparts, yielding improved lattice stability and corrosion resistance.^99^ For instance, PtIrFeCoCu HEI NPs (Fig. 4a) exhibit exceptional durability in ADT tests, showing only a 9 mV negative shift in *E*_1/2_, compared with a 49 mV shift for commercial Pt/C (Fig. 4b), along with negligible compositional change. Density functional theory calculations reveal positive dissolution energies for all constituent elements in HEI NPs, while Fe in pure form shows a negative dissolution energy. This effectively suppresses metal, especially Fe, dissolution (Fig. 4c).^100^ Remarkably, HEI NPs also demonstrate enhanced durability even at ultrasmall particle sizes. While conventional small NPs (<3 nm) typically suffer severe dissolution due to high surface energy, ultrasmall (∼2 nm) PtFeCoNiCuZn HEI NPs remain stable under harsh ORR conditions in fuel cells, exhibiting only a 14.3% loss in power density and negligible degradation after 30 000 cycles at 0.8 A cm^−2^.^101^

Constructing an intermetallic core@Pt shell structure further enhanced the durability against acid. The core@shell configuration is typically achieved by controlled acid leaching of alloy precursors to enrich Pt on the surface. However, the Pt shell must be sufficiently dense and stable to prevent continued metal dissolution during potential cycling.^103^ To this end, post-synthesis thermal annealing is often employed to promote atomic rearrangement and densification of the Pt shell. A representative example is L1_0_-PtCo@Pt, where a defective Pt surface layer generated by dealloying was subsequently annealed to form a compact and continuous Pt shell (Fig. 4d). The resulting NPs exhibited negligible Co loss after 30 000 ADT cycles, highlighting the protective role of the dense Pt shell.^29^ Constructing core@shell architectures on HEA or HEI NPs can further enhance durability by combining the sluggish diffusion effect intrinsic to multicomponent systems with the protective shell barrier, offering outstanding stability under practical fuel-cell conditions. For example, an N-doped PtCoNiFeCu HEI with a ∼1 nm-thick Pt shell effectively prevented the dissolution of early transition metals, even after 90 000 voltage cycles in MEAs. This demonstrates the strong synergistic durability benefits of the HEI core@shell design.^91^

Core@shell structures employing non-Pt cores have also shown potential for improving stability while reducing Pt loading. Deposition of a Pt-based ORR-active shell onto an Au core can greatly enhance resistance to dissolution, as demonstrated in early examples such as Au@FePt_3_.^104^ A more recent study reported a 30-fold reduction in Pt dissolution for a Pt@Au core@shell structure compared with pure Pt NPs of similar size (∼3 nm). The improvement was attributed to the strong Au effect on stabilizing the Pt shell and suppressing surface Pt migration.^20^

A stable core@shell architecture can form *in situ* through surface reconstruction during ORR operation. For example, PtSe_2_ alloys undergo surface activation during extended electrochemical cycling in O_2_-saturated electrolytes: the surface Se atoms are selectively removed, while the remaining Se–Pt bonds in the core stabilize the reconstructed Pt shell, forming a robust PtSe_2_@Pt structure. This self-reconstructed catalyst exhibits minimal activity decay even after 126 000 cycles and remarkable resistance to CO and CH_3_OH poisoning, attributed to the Se-induced weakening of adsorbate binding.^105^ A similar self-reconstruction phenomenon has been observed in PtRh alloys, where the low-redox-potential Rh initially leaches from the surface, leaving Rh vacancies. Concurrently, high-energy Pt atoms dissolve and redeposit into these vacancies, forming a compact “self-healing” Pt layer (Fig. 4e). The resulting PtRh catalyst displays negligible Pt and Rh dissolution after 100 000 ADT cycles.^102^ This concept was further extended to PtCuRh alloys, where the self-healing mechanism effectively suppresses Cu leaching and improves phosphoric acid resistance.^86^

### 3.3 Surface confinement

Coating an NP surface with a robust layer of carbon-based materials, metal oxides, or polymers is an alternative strategy to enhance the durability of Pt-based catalysts. Such a coating can physically shield catalysts from corrosive electrolytes and suppress the migration of poisoning species to the surface, thereby improving resistance to corrosion and poisoning. Moreover, the coating can help trap dissolved metal ions, promoting their redeposition and reducing the overall dissolution rate. The confined layer also serves as a “cage,” anchoring NPs to the support and mitigating aggregation *via* the detach–migrate–attach mechanism or sintering.

**Fig. 5:**
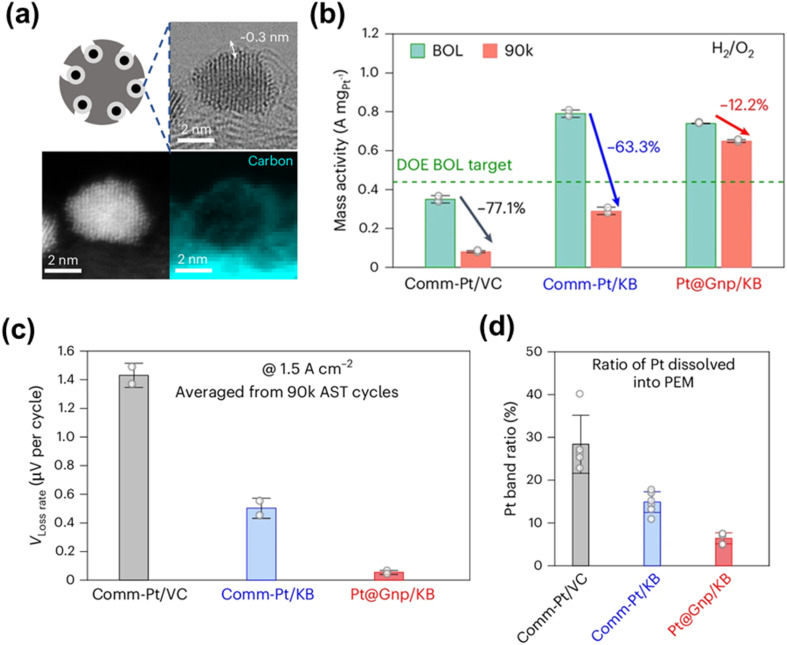
(a) High-resolution scanning TEM and electron energy loss spectroscopy mapping images of Pt@graphene nanopocket on Ketjenblack (Pt@Gnp/KB) catalyst showing that NP is covered by a thin (0.3 nm) carbon layer. (b) Mass activity of commercial Pt supported on Vulcan carbon (Comm-Pt/VC) and supported on Ketjenblack (Comm-Pt/KB), as well as Pt@Gnp/KB evaluated at 0.9 V_*iR*-free_ at the beginning of life (BOL) and end of life (EOL) after 90 000 AST cycles in MEA. (c) Voltage degradation rate (*V*_loss rate_) during square-wave ADT cycling, calculated from the voltage loss at 1.5 A cm^−2^ where the rated power is delivered. (d) Comparison of Pt band ratio, which reflects the degree of Pt dissolution into the PEM. Reproduced from ref. 107 with permission. Copyright © 2025, Springer Nature.

Carbon-based coatings, including graphene and graphitic carbon, have been extensively investigated for improving catalyst stability.^106^ Pt-based NPs were encapsulated within graphene nanopockets *via* an impregnation–annealing method, where metal precursors impregnated on Ketjenblack were rapidly annealed at 200 °C, forming a thin (∼0.3 nm) graphene layer on ∼3 nm Pt NPs (Fig. 5a). This graphene-pocketed Pt catalyst supported on Ketjenblack (denoted as Pt@Gnp/KB) shows strong anti-poisoning ability. The graphene coating reduced sulfonate group coverage to 3–6%, compared to 16% for unprotected Pt, indicating effective mitigation of poisoning. Consequently, the pocketed Pt (Pt@Gnp/KB) retained 87.8% of its mass activity after 90 000 cycles, far exceeding Ketjenblack-supported Pt (36.7%) and showed an order-of-magnitude lower voltage decay in MEA tests (Fig. 5b and c), corresponding to a projected lifetime of 200 000 h, well beyond the DOE target of 30 000 h for heavy-duty vehicles.^107^ Furthermore, Pt@Gnp/KB demonstrated over 50% reduction in Pt dissolution and markedly suppressed aggregation after ADT compared to commercial Pt supported on Ketjenblack (Comm-Pt/KB) (Fig. 5d). Similar graphene-based confinement has been extended to PtM (M = Co, Ni, Fe) alloys.^108^

**Fig. 6:**
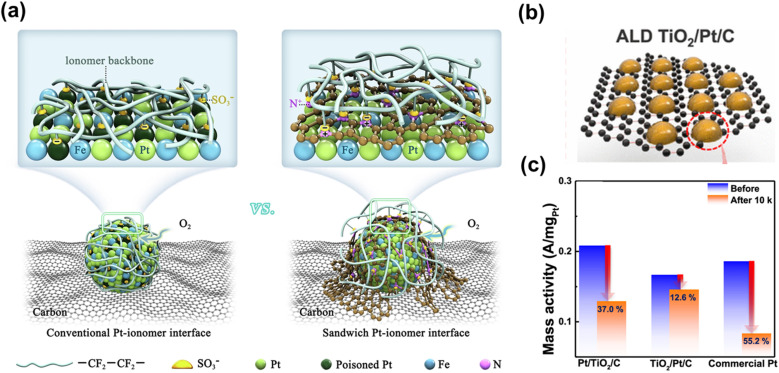
(a) Schematic illustration of N-doped atomically-thin carbon layer in L1_2_-Pt_3_Fe/C shields Pt atoms from ionomer poisoning, increases the number of active sites, and improves O_2_ permeability and long-term stability. Brownish spheres represent N-doped atomically thin carbon layer. Reproduced from ref. 109 with permission. Licensed by CC BY-NC-ND 4.0. (b) Schematic illustration of TiO_2_-coated Pt supported on graphene. (TiO_2_/Pt/C). Black spheres represent the carbon support; brownish spheres represent the TiO_2_-coated Pt NPs. (c) Mass activity at 0.9 V during the initial cycle and after the ADT for Pt/TiO_2_/C, TiO_2_/Pt/C, and commercial Pt/C. Adapted from ref. 111 with permission. Copyright © 2022 American Chemical Society.

Carbon coatings can also mitigate Pt poisoning by ionomer, as demonstrated by porous N-doped amorphous carbon-coated Pt_3_Fe (Fig. 6a). Upon annealing Pt_3_Fe/KB, the Pt_3_Fe phase transforms into an ordered intermetallic structure, while the surface ligand oleylamine is carbonized into an atomic-scale N-doped carbon layer that encapsulates the ordered Pt_3_Fe. The N-doped carbon, derived from oleylamine decomposition, reduced sulfonate coverage on Pt to 10.1% *versus* 21.2% for unprotected Pt, as confirmed by CO displacement experiments. The coating also prevented Fe leaching during ADT, attributed to confinement and Fe–N anchoring effects.^109^ Similarly, micropore-rich carbon coatings derived from Pt-encapsulated MOFs provided excellent tolerance toward phosphoric acid, maintaining morphology after 10 000 cycles and exhibiting superior ORR activity in H_3_PO_4_-containing electrolytes due to steric inhibition of acid adsorption.^110^

Acid-stable metal oxides are also employed to stabilize the Pt catalyst. For instance, TiO_2_-coated Pt catalysts prepared *via* atomic layer deposition demonstrated significantly enhanced durability (Fig. 6b), with only a 12.7% activity loss after 10 000 cycles compared to 55.2% for uncoated Pt (Fig. 6c), attributed to reduced Pt dissolution and aggregation.^111^ TiO_2_ coatings can even protect the carbon support against corrosion in PEMFC environments.^112^ Similarly, SiO_2_ coatings were shown to suppress Pt dissolution and aggregation during ORR.^113^

Perfluorosulfonic acid (PFSA), polytetrafluoroethylene (PTFE), and polyvinylidene fluoride (PVDF) can serve as binders to enhance hydrophobicity and triple-phase boundary formation around Pt-based electrodes.^114,115^ However, such dense polymers may poison Pt or block active sites. Dense polymers like polydopamine can fully encapsulate NPs, compromising their activity even though they effectively protect carbon supports from corrosion and improve Pt dispersion.^116,117^ More recently, polymers with microporosity have been explored as coatings to alleviate phosphoric acid poisoning in high-temperature PEMFCs. The porous structure is engineered to selectively bind phosphate ions without impeding oxygen influx or water efflux. Consequently, Pt utilization was improved from 15% (from traditional PBI-based HT-PEMFCs) to 50%.^118^

Although various encapsulation methods have demonstrated clear advantages in protecting catalysts, they may also impede reactant access to active sites, leading to reduced ECSA and lower ORR mass activity. Nevertheless, well-controlled coating was found not to impact or even enhance ORR activity likely due to improved mass transport facilitated by defective or loosely packed carbon layers.^119^ This observed balance between activity and durability underscores a complex interplay among site accessibility, ionomer adsorption, and mass transport dynamics.

### 3.4 Support materials engineering

The stability of supporting materials is a critical factor in ensuring the durability of catalyst electrodes. To be effective, these supporting materials must first overcome the inherent limitations of conventional carbon black, which is susceptible to corrosion under the acidic and oxidative conditions typical of PEMFC environments. An ideal support should exhibit a strong binding affinity to NP catalysts to prevent their detachment. Additionally, it should enable tunable NP–support interactions that moderate the binding strength of oxidative and poisonous species. This controlled interaction enhances catalyst durability by mitigating both metal dissolution and catalyst poisoning.

Carbon materials are widely used as supports in catalyst systems. Among them, carbon black has seen extensive commercial applications. Efforts to enhance the stability of support materials have led to the development of more robust alternatives, including graphitized carbon black, mesoporous carbon, carbon nanotubes (CNTs), graphene, and its derivatives such as graphene oxide (GO) and reduced graphene oxide (rGO).^122,123^ Mesoporous carbon, rich in pore structures, can effectively accommodate Pt NPs, preventing direct ionomer coverage on Pt while facilitating the transport of O_2_ and H_2_O. This configuration significantly boosts catalytic activity by alleviating poisoning effects. Furthermore, the confinement within the pores inhibits the migration and aggregation of Pt NPs, enhancing structural stability.^123^ Compared with defect-rich carbon blacks, where oxidation often initiates at defect sites,^124^ graphitized carbons with higher crystallinity contain fewer defects and thus exhibit enhanced corrosion resistance.^125^ Pt supported on graphitized carbon shows only 24% performance loss, compared with 54% for Pt/carbon black, primarily due to reduced particle aggregation.^126^ Similarly, graphene possesses a highly ordered sp^2^-hybridized framework that provides excellent anti-corrosion stability. However, its chemical inertness limits NP deposition, requiring oxidation or reduction treatments to produce GO or rGO with tunable defect density and improved conductivity. Pt NPs supported on rGO exhibit superior durability, maintaining ORR activity even after 10 000 ADT cycles, owing to the strong anchoring of Pt to defect sites. CNTs also demonstrate better electrochemical stability than carbon black, with 30% lower corrosion current and less surface oxide formation under PEMFC operation.^127^ As with graphene, CNTs typically require surface functionalization to improve NP attachment.

Non-carbon-based supports mainly consist of metal oxides such as SnO_2_ and TiO_2_, which exhibit strong metal–support interactions that help anchor Pt NPs and prevent aggregation.^128^ However, despite their excellent resistance to oxidative corrosion,^129^ these oxide supports typically suffer from poor electrical conductivity, limiting their use as stand-alone supports. To overcome this, they are often doped or composited with conductive carbon materials, thereby balancing conductivity and durability.^130^

**Fig. 7:**
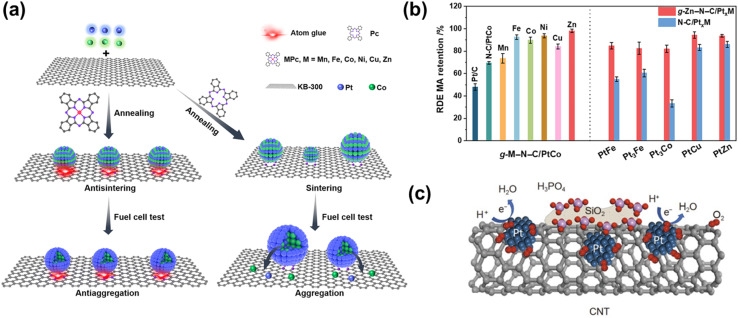
(a) Schematic diagram of the preparation process of L1_0_-PtCo supported on M–N–C ‘atomic glue’ and structural evolution of carbon-supported L1_0_-PtCo with/without atomic glue modification. (b) Mass activity retention of PtM NPs supported on M′–N–C (M = Fe, Co, Cu, Zn, M′ = Mn, Fe, Co, Ni, Cu, Zn) after 10 K voltage cycles ADT in a rotating disk electrode setup. Reproduced from ref. 120 with permission. Copyright © 2024, The American Association for the Advancement of Science. (c) Schematic illustration of Pt NPs supported on SiO_2_-coated CNTs to resist phosphoric acid poisoning. Reproduced from ref. 121 with permission. Copyright © 2021, Science China Press and Springer-Verlag GmbH Germany, part of Springer Nature.

Further modification of supports involves heteroatom or metal doping to enhance both stability and metal–support interaction. For instance, fluorine (F) doping increased carbon framework stability by 50–80% due to the passivation effect of F, and also enhanced hydrophobicity, which suppresses Pt oxide formation and subsequent Pt dissolution.^131^ Pyrrolic-N species may also enhance Pt oxidative stability.^132^ N doping primarily benefits catalyst durability through the formation of strong Pt–N–C interactions, which suppress NP detachment and dissolution.^22^ Incorporating transition metals (Zn, Mn, Fe, Ni, Co, Cu) along with N to form M–N–C single-atom sites further boosts catalyst durability (Fig. 7a).^120^ For example, Zn–N–C acted as an “atomic glue” to anchor L1_0_-PtCo NPs strongly, resulting in only 7.9% mass activity loss after 30 000 cycles, compared with 53.3% for Pt/C and 48.4% for N–C/PtCo (Fig. 7b). This enhanced stability enabled steady-state operation for over 230 hours at 1.5 mA cm^−2^ in practical fuel cells. Similar improvements have been observed for other L1_0_-PtM/M–N–C (M = Fe, Co, Ni) systems.^133^ However, doping can also compromise stability if the dopant atom introduces lattice strain or defects. The atomic radius of the dopant should not deviate significantly from that of carbon, as excessive geometric distortion can reduce durability.^134^ For instance, O_2_ was found to preferentially adsorb near N sites in N-doped CNTs, promoting oxidation,^135^ while pyridinic-N dopants deteriorated support stability by generating N-related defect sites.^136^

Finally, composite supports have been developed to combine the advantages of multiple materials or to mitigate their individual drawbacks. For instance, 2D rGO sheets tend to restack and block active sites, hindering mass transport. Introducing carbon black spacers between rGO layers prevented restacking and led to composite supports that retained over 80% of their initial ECSA after ADT—superior to Pt supported on either carbon black or rGO alone.^137^ Similarly, metal oxide/carbon composites such as Pt/Y_2_O_3_/C, Pt/Gd_2_O_3_/C,^138^ Pt/CeO_2_/C,^139^ and Pt/WO_3_/C^140^ can enhance Pt NP anchoring and improve the NP durability. Composite architectures can also alleviate catalyst poisoning; for example, SiO_2_-coated CNTs were shown to adsorb phosphoric acid more strongly than Pt, thereby preventing acid poisoning of the Pt sites (Fig. 7c).^121^

## 4 Conclusions and outlooks

In this perspective, we highlight that the long-term stability of Pt-based catalysts remains a major challenge impeding the widespread commercialization of PEMFCs. Various degradation pathways, including metal dissolution, particle aggregation, support corrosion, and surface poisoning, collectively compromise catalytic activity and durability. Recent advances have introduced diverse strategies to enhance the resilience of Pt-based cathodes, such as alloying and doping, structural engineering, surface confinement, and support material optimization. Notable progress has been made in elucidating degradation mechanisms and developing stabilization approaches at both the material and structural levels, paving the way toward durable and efficient catalysts for next-generation fuel cells.

A key research direction will be to elucidate the precise surface structure of Pt-based catalysts and their dynamic structural evolution during fuel cell reactions. Advanced characterization techniques, particularly *in situ* and *operando* methods, will play a pivotal role in this pursuit. The advent of four-dimensional scanning transmission electron microscopy (4D-STEM) enables direct visualization of the dynamic evolution of Pt catalysts under realistic reaction conditions, providing critical insights into the origins of their stability changes during operation.^141^ Concurrently, *in situ* synchrotron-based techniques such as extended X-ray absorption fine structure (EXAFS) and X-ray absorption near-edge structure (XANES), coupled with time-resolved spectroscopy,^142^ offer valuable information on oxidation state evolution, local coordination environments, and transient Pt–O intermediates formed during fuel cell operations. *Operando* X-ray Computed Tomography (XCT),^143^ Small-Angle X-ray Scattering (SAXS),^144,145^ and Neutron Imaging^146^ can provide real-time insights into particle aggregation and catalyst layer evolution. These techniques are essential for elucidating catalyst degradation mechanisms across both atomic and micron scales.

Computational chemistry provides another indispensable approach to uncovering electrode reaction mechanisms. First-principles calculations can offer atomistic insight into reaction pathways and guide the rational design of more stable catalysts. The integration of high-throughput screening with machine learning (ML) may further accelerate materials discovery by drastically reducing experimental trial cycles, particularly in the rapidly emerging field of high-entropy alloys.^147^ The convergence of computation, ML, and experiment will thus be a powerful strategy for identifying next-generation Pt-based catalysts with enhanced anti-poisoning capability and long-term operational stability.

From a design standpoint, the strategies discussed in this work—such as alloying, intermetallic structuring, surface confinement, and support engineering—will remain central. Future efforts are expected to combine multiple synthesis approaches to harness their synergistic effects. For instance, core@shell intermetallic Pt catalysts may simultaneously exhibit the high intrinsic activity of ordered alloys and the enhanced stability derived from surface protection against transition-metal leaching. Hybrid systems integrating Pt with non-noble-metal or single-atom catalysts also hold promise for lowering cost while maintaining high performance.^148^ Beyond catalyst architecture, the development of scalable and environmentally benign synthesis routes will be vital for practical implementation.

From an industrial perspective, while LT-PEMFCs remain the primary focus, alternative HT-PEMFCs employing polybenzimidazole (PBI) membranes or modified perfluorosulfonic acid membranes are actively being explored to enable higher operating temperatures.^149^ These elevated temperatures can simplify thermal and water management, but also introduce significant challenges to the stability of Pt-based cathode catalysts. To address these issues, research should prioritize the development of acid-tolerant and poison-resistant Pt catalysts, particularly for conditions exceeding 100 °C in the presence of phosphoric acid or its derivatives. Additionally, standardized protocols for durability testing under such high-temperature environments are urgently needed. Enhancing sustainability and cost-efficiency also calls for innovations in catalyst recycling and reactivation. Environmentally benign leaching agents are being investigated for Pt recovery from spent membrane electrode assemblies, while techniques such as *in situ* electrochemical redispersion, atomic layer deposition, and support-driven self-healing offer promising routes for regenerating Pt NPs with restored dispersion and optimized electronic properties.

Looking ahead, process optimization will be key to the commercialization of catalysts, while novel catalyst design will be crucial for overcoming the scientific challenges encountered thus far. AI-driven approaches are expected to make future research more data-informed, efficient, and sustainable, and will undoubtedly accelerate the discovery of new catalyst systems for commercial PEMFC applications.

## Author contributions

Yuliang Chen, Linghang Meng, and Haobo Sun contributed to the conceptualization and drafting of the manuscript. All authors contributed to manuscript revision. Shouheng Sun was responsible for funding acquisition, project administration, and writing finalization.

## Conflicts of interest

The authors declare no conflicts of interest.

## Data Availability

No primary research results, software or code have been included and no new data were generated or analysed as part of this review.
